# Tracking the Response to Immunotherapy: Blood microRNA Dynamics in Patients With Advanced Non–Small Cell Lung Cancer

**DOI:** 10.1200/PO-24-00790

**Published:** 2025-05-22

**Authors:** Maria Vittoria Chiaruttini, Claudia Proto, Giuseppe Lo Russo, Arsela Prelaj, Miriam Segale, Anna Zanghì, Francesca Galli, Francesca G. Greco, Diego Signorelli, Marta Brambilla, Mario Occhipinti, Filippo De Braud, Marina C. Garassino, Gabriella Sozzi, Eliana Rulli, Mattia Boeri

**Affiliations:** ^1^ Laboratory of Methodology for Clinical Research, Clinical Oncology Department, Istituto di Ricerche Farmacologiche Mario Negri IRCCS, Milan, Italy; ^2^ Unit of Biostatistics, Epidemiology and Public Health, University of Padua, Padua, Italy; ^3^ Thoracic Oncology Unit, Department of Medical Oncology, Fondazione IRCCS Istituto Nazionale dei Tumori, Milan, Italy; ^4^ Department of Electronics, Information, and Bioengineering, Polytechnic University of Milan, Milan, Italy; ^5^ Epigenomics and Biomarkers of Solid Tumors Unit, Experimental Oncology Department, Fondazione IRCCS Istituto Nazionale dei Tumori, Milan, Italy; ^6^ Department of Radiology, Fondazione IRCCS Istituto Nazionale dei Tumori, Milan, Italy; ^7^ Niguarda Cancer Center, Grande Ospedale Metropolitano Niguarda, Milan, Italy; ^8^ Section of Hematology-Oncology, Department of Medicine, Knapp Center for Biomedical Discovery, The University of Chicago, Chicago, IL

## Abstract

**PURPOSE:**

Despite the significant improvement in outcomes for patients with advanced non–small cell lung cancer (NSCLC) treated with immune checkpoint inhibitors (ICIs), resistance, whether primary or secondary, remains a substantial challenge. Currently, reliable biomarkers to monitor ICI response are lacking, highlighting the need for minimally invasive tools like liquid biopsy to track treatment efficacy. This study aimed to identify circulating microRNAs (miRNAs) as potential biomarkers to track ICI response in patients with NSCLC.

**MATERIALS AND METHODS:**

The Apollo longitudinal study enrolled patients with advanced NSCLC receiving ICI in first or subsequent lines. Plasma samples were collected at baseline and follow-up to prospectively assess miRNA profiles until progressive disease (PD). Using a custom reverse transcription-quantitative polymerase chain reaction platform, 276 ratios among 24 lung cancer–related miRNAs were analyzed. The generalized estimating equation and joint models were applied to select the miRNA ratios most associated with PD over time. To control for multiple testing, the Benjamini-Yekutieli method was applied setting a 10% false discovery rate threshold.

**RESULTS:**

From the 211 patients, a total of 454 plasma samples were analyzed. Clinical and biochemical variables had little effect on miRNAs' profile. The analysis identified nine miRNA ratios, all involving miR-145-5p, as significant biomarkers for monitoring treatment response, even after adjustment for the line of therapy. These ratios exhibited a longitudinal modulation pattern consistent with radiologic response, particularly in patients who initially benefited from ICI treatment. In addition, in an independent set of 32 plasma samples from 10 patients receiving ICI as maintenance therapy, the same trends were observed.

**CONCLUSION:**

A focused panel of miRNA ratios, driven by miR-145-5p, effectively reflects response to ICI therapy in patients with advanced NSCLC, highlighting their potential as biomarkers for treatment monitoring.

## INTRODUCTION

Treatment with immune checkpoint inhibitors (ICIs), targeting PD-1/PD-L1, drastically prolonged overall survival (OS) in patients with non–small cell lung cancer (NSCLC).^1-3^ However, about 80% of patients ultimately relapse because of primary or secondary resistance.^4^ To date, PD-L1 expression by tumor cells and tumor mutation burden (TMB) are the only biomarkers commonly used in clinical practice to define the best immunotherapeutic strategy in advanced NSCLC.^5-7^ Despite an established predictive role of these tumor tissue biomarkers, the power in selecting patients is still limited and the comprehension of the mechanisms underlying tumor responsiveness to ICIs is incomplete.^8^

**CONTEXT**

**Key Objective**
To explore the potential of circulating microRNAs (miRNAs) as biomarkers for monitoring treatment response in patients with advanced non–small cell lung cancer (NSCLC) receiving immune checkpoint inhibitors (ICIs).
**Knowledge Generated**
The study identified a set of miRNA ratios, particularly those involving miR-145-5p, whose longitudinal modulation was associated with the radiologic response in first and further lines of therapy and in maintenance therapy. These findings suggest that miRNA-based liquid biopsies can provide real-time insights into treatment efficacy.
**Relevance**
This miRNA profiling approach offers a minimally invasive and easy-to-use method to monitor NSCLC patient response to ICIs, potentially enabling clinicians to tailor therapeutic strategies and make timely adjustments to improve patient outcomes.

Nowadays, it is becoming clear that additional tumor-intrinsic, tumor-extrinsic, and host-related factors might affect response to ICIs.^9,10^ Among others, the presence of driver mutations,^11^ a pre-existing CD8^+^ T-cell infiltrate,^12^ the T-cell receptor (TCR) clonality,^13^ and the loss of the human leukocyte antigen class I locus have been tested as predictive biomarkers of response to ICIs at baseline (BL).^14^ With the advent of liquid biopsies, blood TMB at BL and the dynamics of circulating tumor DNA (ctDNA) throughout treatment were associated with the response to ICIs in many cancer types.^15-18^ The identification of biomarkers that are easy to measure using minimally invasive techniques could thus be useful to monitor the response to treatment.

Thanks to the Apollo observational longitudinal study, we collected real-world data about patients with advanced NSCLC treated with ICIs in our institute. From enrolled patients, clinical, biochemical, radiologic, and molecular data and plasma samples were collected at BL and during follow-up. In this context, we paid particular attention to circulating microRNAs (miRNAs). Indeed, these small molecules serve as epigenetic regulators implicated in numerous mechanisms of therapy resistance within both the tumor and the tumor microenvironment (TME).^19,20^ Encapsulated in extracellular vesicles, miRNAs also play a role as mediators of cell-to-cell communication and can be reliably quantified through liquid biopsies.^21-23^

Plasma samples were prospectively profiled using a custom panel of 24 lung cancer–related circulating miRNAs. Functional studies have shown how these miRNAs are able to promote tumor growth and reflect the switch toward a protumorigenic or immunosuppressive profile of stromal cells such as fibroblast, neutrophils, lymphocytes, and macrophages.^24-28^ Moreover, analysis at the BL already showed that a miRNA signature classifier was associated with the survival of patients with advanced NSCLC independent of PD-L1 expression or clinical characteristics.^29,30^

Here, we report the final results of plasma miRNA profiling in 454 samples longitudinally collected at fixed time points from patients with 211 NSCLC. Molecular and radiologic data were combined to define a mathematical model for the identification of features associated with the probability of disease progression over time.

## MATERIALS AND METHODS

### Patients' Enrollment, Follow-Up, and Timing of Sample Collection

From 2015 to 2023, on signing the informed consent, the Apollo observational and longitudinal study enrolled and followed patients with advanced NSCLC treated with ICIs for the assessment of multiple markers of response to treatment (details in the Protocol). Clinical and biochemical variables including sex, age, smoking status, line of therapy, Eastern Cooperative Oncology Group performance status, histology, liver metastasis, PD-L1 tumor expression, neutrophil-lymphocyte ratio, leukocytes, monocytes, and lactate dehydrogenase were collected at BL. Plasma samples for miRNA profiling and radiologic information were also collected at BL and during treatment until progressive disease (PD). This study complied with the Declaration of Helsinki, was approved by the Ethics Committee of the Fondazione IRCCS Istituto Nazionale dei Tumori of Milan (code: INT_22/15), Italy, and adheres to the REMARK criteria as listed in their guidelines.^31^

### Radiologic Assessment of the Response to Treatment

Radiologic tumor assessment was performed every 9 weeks (±2 weeks) or more frequently, if clinically indicated. The RECIST version 1.1 was adopted to measure patients' tumor response to immunotherapy.^32^ Images were analyzed using the Syngo-plaza software (Siemens Healthcare, Forchheim, Germany).

### Plasma Sample Collection and microRNA Profiling

Plasma was separated within 2 hours from blood sample collection to minimize the hemolysis, as previously described.^33^ The miRNA profile was assessed using custom TaqMan Array MicroRNA Cards (Thermo Fisher Scientific, Waltham, MA) or miRCURY LNA plates (Qiagen, Hilden, Germany) with proper controls and following standard operating procedures.^34^ Details are provided in Appendix 1.

### Definition of miRNA Ratios for Computational Analysis

Data were processed using the ViiA7 RUO software (Thermo Fisher Scientific), and background signal was removed setting automatic BL cycles and a fixed threshold of 0.15 for all miRNAs. Computational analyses were conducted considering the pairwise ratios of variables (miRNA ratios). The mean cycle threshold (Ct) value of the two duplicates was considered for the analysis. Since Ct values are in a binary logarithmic scale and inversely correlate with the miRNA amount, the ratio between couples of miRNAs was calculated as the additive inverse ΔCt value (ie, miR-x/miR-y = CtmiR-y – CtmiR-x). Overall, 276 miRNA ratios were generated and used for the analysis.

### Statistical Analysis

#### Description of the Population Characteristics

Descriptive statistics for the available clinical features such as absolute frequencies (percentages) and means (standard deviation) or medians (IQR) for categorical and continuous variables, respectively, were provided. To evaluate the association between BL clinical features and both progression-free survival (PFS) and OS, the univariate Cox regression model was applied. The hazard ratios and the corresponding 95% CIs were reported.

#### Description of miRNA Ratios and Their Association With Clinical Features

Unsupervised hierarchical clustering of miRNA ratios and samples was performed by centered one minus correlation and an average linkage approach to evaluate whether technical aspects such as batch effect, year and time of collection, and levels of hemolysis could affect the molecular analysis. The analysis was conducted using BRB-Array Tool v.4.6.2 (National Cancer Institute, Rockville, MD) and the Cluster Analysis of Genes and Samples function to create the heatmap.

In addition, we conducted principal component (PC) analysis on the 276 miRNA ratios using the FactoMineR R package (R Core Team, Vienna, Austria). This allowed us to assess the relationship according to the Spearman correlation test between each PC and various BL clinical features, shedding light on how these variables might influence plasma miRNA profiling. The two-sample *t* test was adopted for class comparison analysis to define miRNA ratios' signatures associated with BL clinical features. For continuous clinical variables, classes were separated on the median value. To account for multiple testing under dependency and control the type I error rate, we applied the Benjamini-Yekutieli (BY) method to adjust *P* values and features with a *P* value of <.05 and a false discovery rate (FDR) threshold of 10% were considered significant. This approach is particularly suitable when tests are correlated, as in our study, where each miRNA is present in multiple ratios.^35^

#### Progression Probability Models Through Longitudinal miRNA Ratio Samples

To identify suitable candidates among the 276 available miRNA ratios, we first selected the straightforward and effective generalized estimating equation (GEE) model, tailored for binary responses.^36^ GEE is a population-averaged approach that estimates how time-dependent covariates, such as miRNA ratios, influence the average outcome over time while accounting for correlations within individual responses. To assess the independent association between each miRNA ratio and the probability of progression, we adjusted the models for both the time to blood sample collection and the BL values of the miRNA ratios. We selected an autoregressive correlation structure with a lag of 1 as this configuration yielded the lowest quasi-likelihood under the independence model criterion, indicating a superior model fit compared with other correlation structures.^37^ From each of the 276 models, we extracted the *P* value and the q value corresponding to the effect of the miRNA ratio.

To further refine the selection of miRNA ratios, we used advanced joint models. These models explicitly account for the dependency between longitudinal changes in miRNA ratios and event-time data, such as PFS. This approach is particularly suitable in cases of nonrandom dropout, where the probability of dropout depends on unobserved longitudinal data, as observed in our cohort.^38^ To account for varying BL hazards among patients undergoing different lines of therapy, we incorporated the line of therapy as a covariate in our joint longitudinal time-to-event models. Finally, we extracted from each model the *P* value and q value corresponding to the estimated association between miRNA ratio and time to progression.

To control for multiple testing, we adjusted all *P* values using the BY method and features with a *P* value of <.05 and a 10% FDR were considered significant. All analyses were conducted using R version 4.4.2 (R Core Team, Vienna, Austria), released on October 31, 2024.

## RESULTS

### Patients' Characteristics and Follow-Up

**FIG 1.**
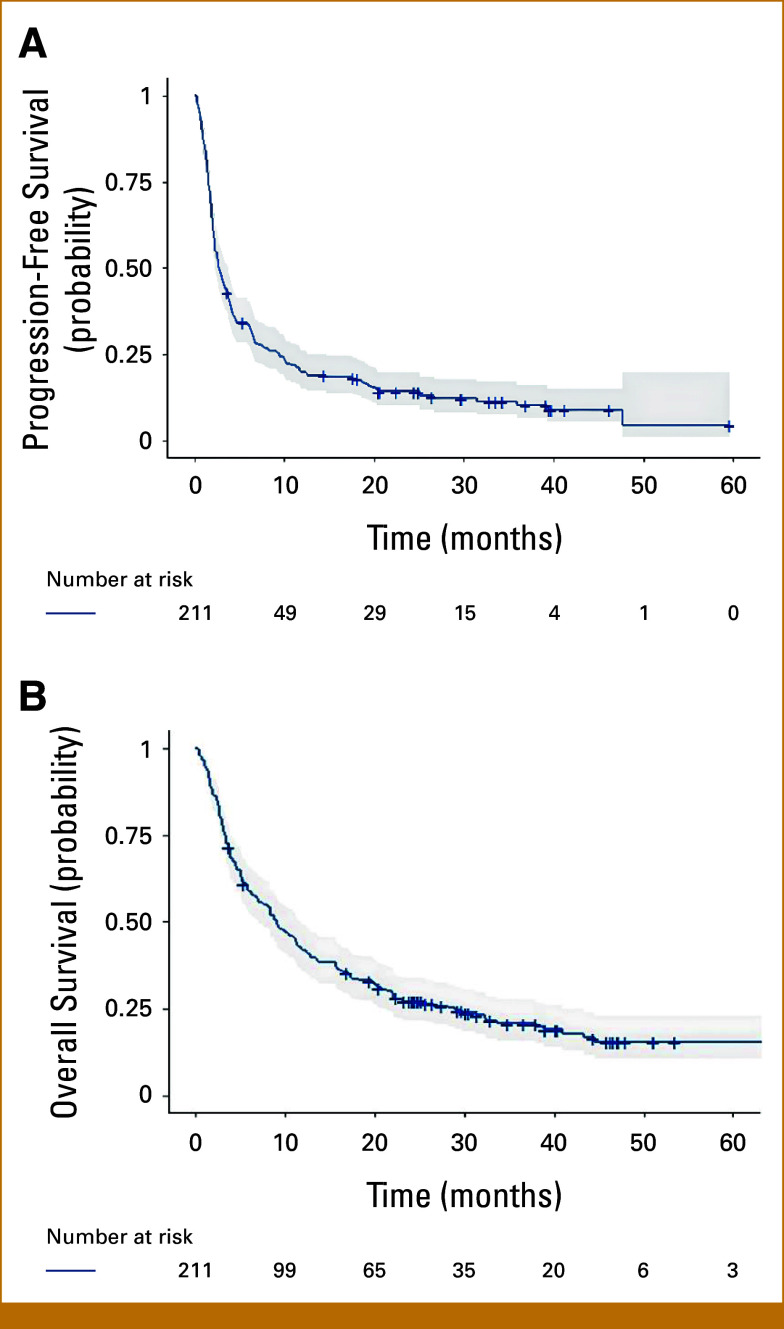
Kaplan-Meier plot for (A) progression-free survival and (B) overall survival. Shadows represent the 95% CIs.

**FIG A1.**
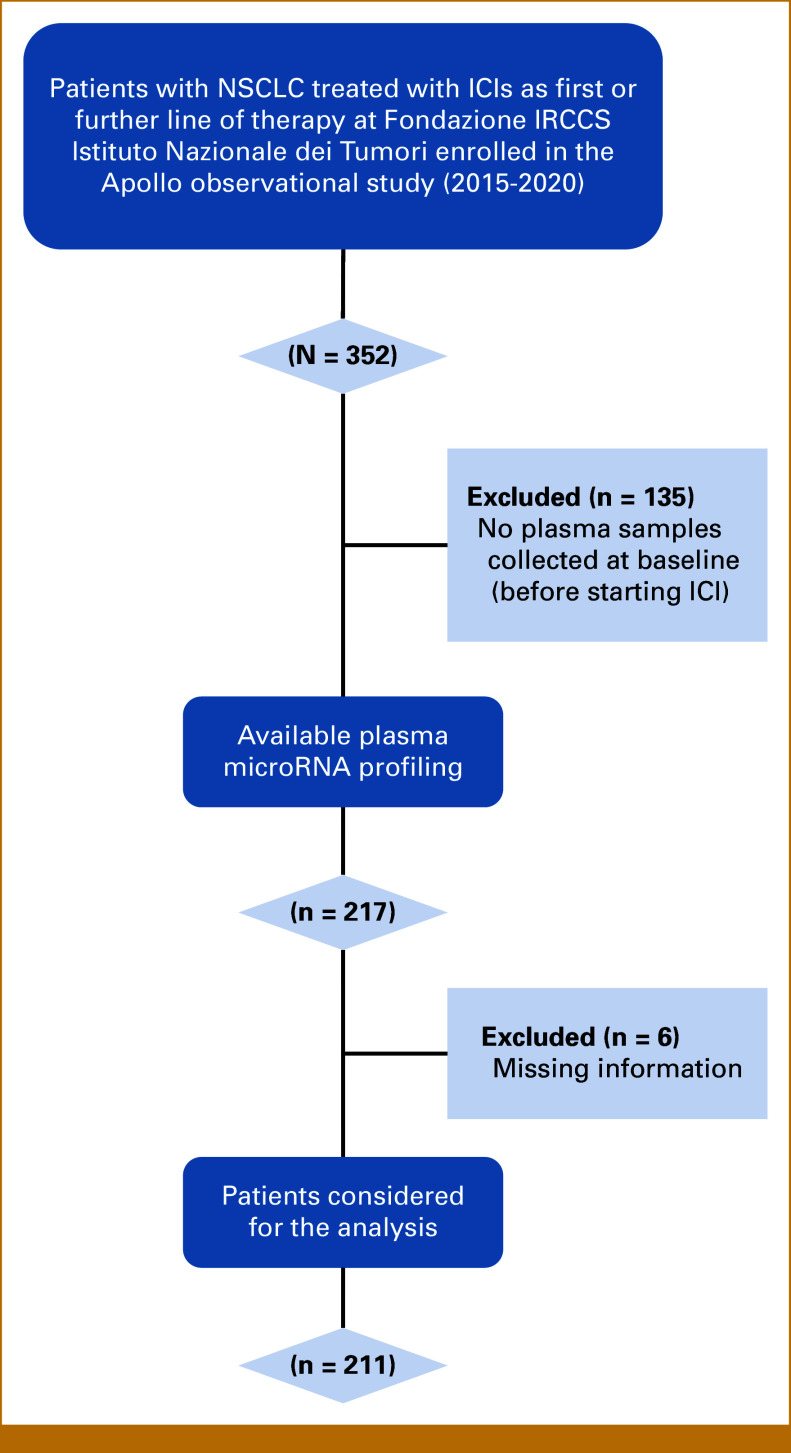
CONSORT diagram describing the Apollo study population considered for the present study. ICI, immune checkpoint inhibitor; NSCLC, non–small cell lung cancer.

In the present study, 211 consecutive patients with advanced NSCLC receiving ICI in first or subsequent lines of therapy were included (Appendix Fig A1). The median follow-up was equal to 39 (95% CI, 32.8 to 46.3) months. Overall, the median PFS and OS were 2.7 (95% CI, 2.3 to 3.8) and 9.1 (95% CI, 7 to 11.7) months, respectively (Fig 1). The total number of PFS events was equal to 186 with 25 censorships, whereas the number of deaths was 166 with 45 censorships. The clinicopathologic characteristics of patients and univariate associations with PFS and OS are reported in Table 1.

**TABLE 1.** Clinicopathologic Characteristics of Patients and Univariate Associations (Cox models) With OS and PFS

Variables	No. (%)	Univariate Analysis, OS		Univariate Analysis, PFS	
		HR (95% CI)	*P*	HR (95% CI)	*P*
Sex			.6		.9
Male	131 (62.1)	Ref		Ref	
Female	80 (37.9)	0.93 (0.67 to 1.27)		0.97 (0.73 to 1.32)	
Age, years, mean (SD)	66.6 (9.5)	1.01 (0.99 to 1.019)	.5	1.005 (0.99 to 1.019)	.5
Smoking			.4		.5
Never	29 (13.7)	Ref		Ref	
Former/current	182 (86.3)	0.84 (0.55 to 1.27)		0.88 (0.59 to 1.31)	
Line of therapy			**<.0001**		**<.0001**
1	78 (37)	Ref		Ref	
>1	102 (48.3)	2.35 (1.67 to 3.31)		1.88 (1.38 to 2.56)	
Performance status			**<.0001**		**<.0003**
0-1	181 (85.8)	Ref		Ref	
2	30 (14.2)	3.13 (2.07 to 4.74)		2.13 (1.42 to 3.20)	
Liver metastasis			**.005**		**.003**
No	37 (17.5)	Ref		Ref	
Yes	174 (82.5)	1.73 (1.18 to 2.52)		1.74 (1.21 to 2.51)	
Histology			**.006**		**.0452**
Squamous	48 (22.8)	Ref		Ref	
Nonsquamous	163 (77.3)	0.6 (0.43 to 0.87)		0.71 (0.51 to 0.99)	
PD-L1 expression			**.0003**		**<.0001**
<50%	110 (62.9)	Ref		Ref	
≥50%	65 (37.1)	0.49 (0.34 to 0.72)		0.41 (0.29 to 0.59)	
Missing	36				
Neutrophil-lymphocyte ratio, median (IQR)	3.7 (2.2-5.8)	1.06 (1.04 to 1.08)	**<.0001**	1.07 (1.05 to 1.10)	**<.0001**
Leukocytes, 10/μL, median (IQR)	8.2 (6.3-11.2)	1.06 (1.04 to 1.09)	**<.0001**	1.04 (1.02 to 1.06)	**<.0001**
Missing	2				
Monocytes, 10/μL, median (IQR)	0.5 (0.4-0.6)	2.81 (1.86 to 4.25)	**<.0001**	1.97 (1.33 to 2.93)	**.0008**
Missing	2				
Lactate dehydrogenase, U/L, median (IQR)	362 (310-444)	1.53 (1.03 to 2.27)	**.04**	1.33 (0.91 to 1.96)	.1
Missing	1				

NOTE. Bold indicates *P* < .05.

Abbreviations: HR, hazard ratio; OS, overall survival; PFS, progression-free survival; SD, standard deviation.

For molecular analysis, we considered samples collected at BL and fixed time points, including the first radiologic examination (R1) and yearly follow-up (Y1, Y2 and Y3), until the time of PD. Whenever feasible, samples collected at the time of the last radiologic examination before PD were also included.

### Biochemical and Clinical Variables Associated With the Circulating microRNA Profile

**FIG 2.**
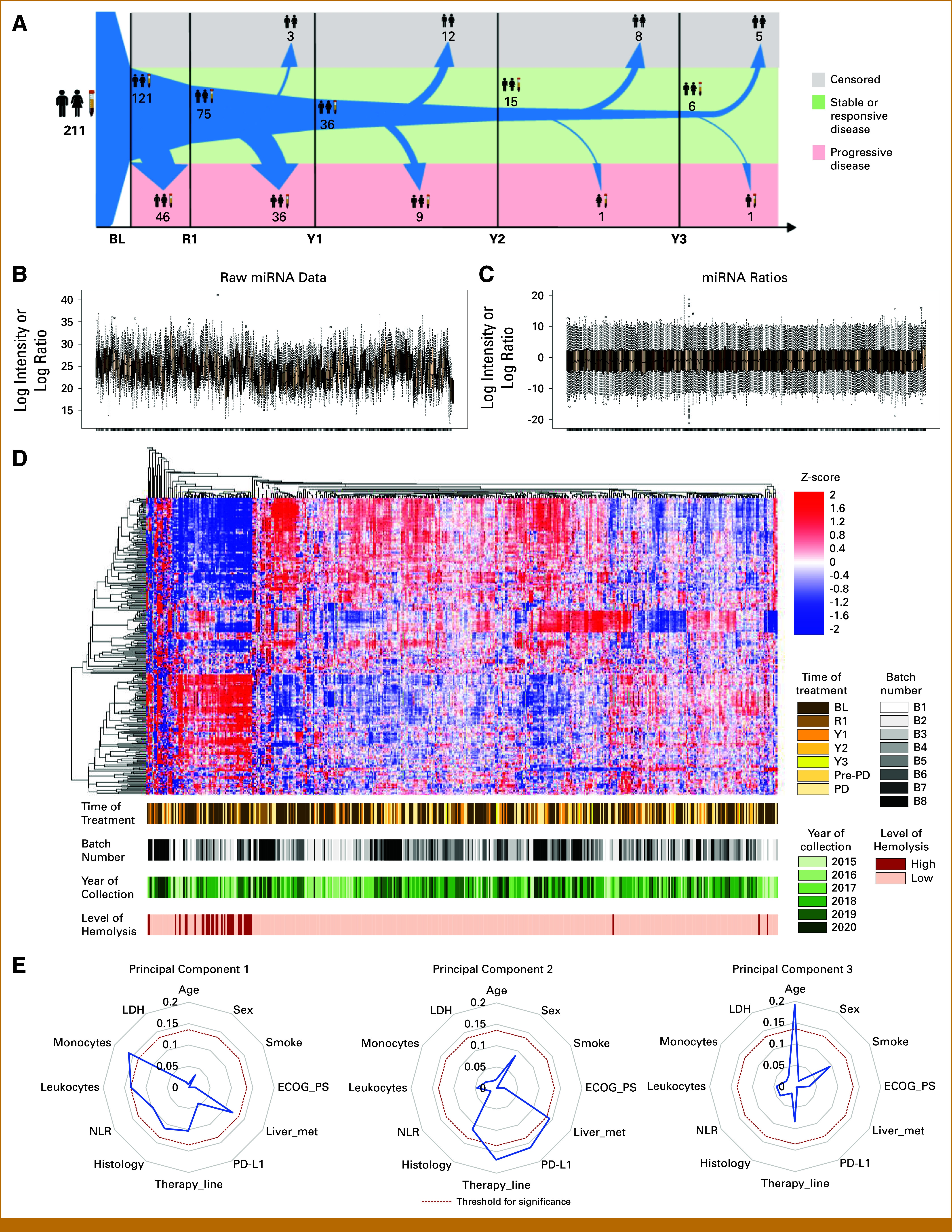
Over-time plasma sample collection and circulating miRNA profiling. (A) Map of samples collected at BL, first radiologic examination (R1), and subsequent years of follow-up (Y1, Y2, Y3) from the 211 patients with NSCLC overall and the 121 considered for longitudinal analysis. Arrows' size reflects the number of available plasma samples. Green background is for samples collected while patients benefit from ICI treatment (stable or responsive disease). Red background is for samples collected at the time of PD. Gray background is for censored patients. Box plot reporting the distribution of values (B) before and (C) after miRNA ratio transformation in each plasma sample. (D) Hierarchical unsupervised clustering analysis considering 450 samples with available molecular profiling and the 276 unique miRNA ratios generated. Samples were classified in strata of characteristics such as batch number, year of sample collection, and time of treatment: BL, R1, Y1, Y2, Y3, PD, and last radiologic examination before PD. (E) Radar plots reporting the association, as by the Spearman correlation test, of BL clinical variables with the first three PCs derived from PC analysis of plasma miRNA profiling. BL, baseline; ECOG, Eastern Cooperative Oncology Group; ICI, immune checkpoint inhibitor; LDH, lactate dehydrogenase; miRNA, microRNA; NLR, neutrophil-lymphocyte ratio; NSCLC, non–small cell lung cancer; PC, principal component; PD, progressive disease; PS, performance status.

During the whole study, 454 plasma samples were profiled for the 24 lung cancer–related miRNAs (Fig 2A). Four of the 454 (0.9%) samples analyzed technically failed the molecular profiling, and high hemolysis levels were measured in 34 (7.5%) samples. To analyze data, we first applied the miRNA ratio transformation generating 276 features. Since we cannot control the exact amount of starting material for each individual sample (ie, the percentage of miRNA relative to the total RNA extracted), this step was necessary to correct the intersample variability (Figs 2B and 2C). By unsupervised clustering analysis, samples with high levels of hemolysis tend to group together, whereas no batch effect or any other clustering because of sampling procedures (ie, year of collection) or sample characteristics (ie, treatment time) was observed (Fig 2D).

To assess the possible relation between clinical variables and the circulating miRNA profile, we start running a PC analysis with BL data. PC1, PC2, and PC3 (which explain 52.6% of the total variance) were slightly but significantly associated with the monocyte and leukocyte count, line of therapy, PD-L1, liver metastases, and patient age (Fig 2E). Moreover, when comparing miRNA profiles of patients stratified based on clinical variables, specific miRNA ratio signatures were identified only for the line of therapy and liver metastases (Appendix Tables A1 and A2).

### Modulation of microRNAs During Treatment With ICI

To model the probability of progression based on longitudinal changes in miRNA ratios, we focused on 121 patients with NSCLC with a total of 360 plasma samples collected over time. Among the 276 miRNA ratios analyzed, 18 were significantly associated with PD according to the GEE model (Appendix Table A3). Of these, nine ratios (including miR-145-5p in either the numerator or the denominator) remained significant in the adjusted joint model, independent of the line of therapy (Table 2).

**TABLE 2.** MicroRNA Ratios Associated With Progression-Free Survival, According to the Joint Model Adjusted by the Line of Therapy, Estimated in the Whole Cohort of 121 Patients With Lung Cancer With Longitudinal Data Treated With Immune Checkpoint Inhibitors

MicroRNA Ratio	Sample Size (events)	α1	SE	*P*	q Value
miR-101/miR-145	117 (88)	–0.638	0.2392	.0076	0.0683
miR-140-5p/miR-145	121 (93)	–0.4968	0.1528	.0012	0.0236
miR-145/miR-15b	121 (93)	1.7072	0.4958	.0006	0.0189
miR-145/miR-17	121 (93)	1.5143	0.5677	.0076	0.0683
miR-145/miR-30b	121 (93)	0.9387	0.3817	.0139	0.0972
miR-145/miR-30c	121 (93)	1.4396	0.3678	.0001	0.0063
miR-145/miR-451	121 (93)	0.3672	0.1411	.0093	0.0731
miR-145/miR-660	120 (92)	0.4135	0.1303	.0015	0.0236
miR-145/miR-92a	121 (93)	0.713	0.2663	.0074	0.0683

Abbreviation: SE, standard error.

**FIG 3.**
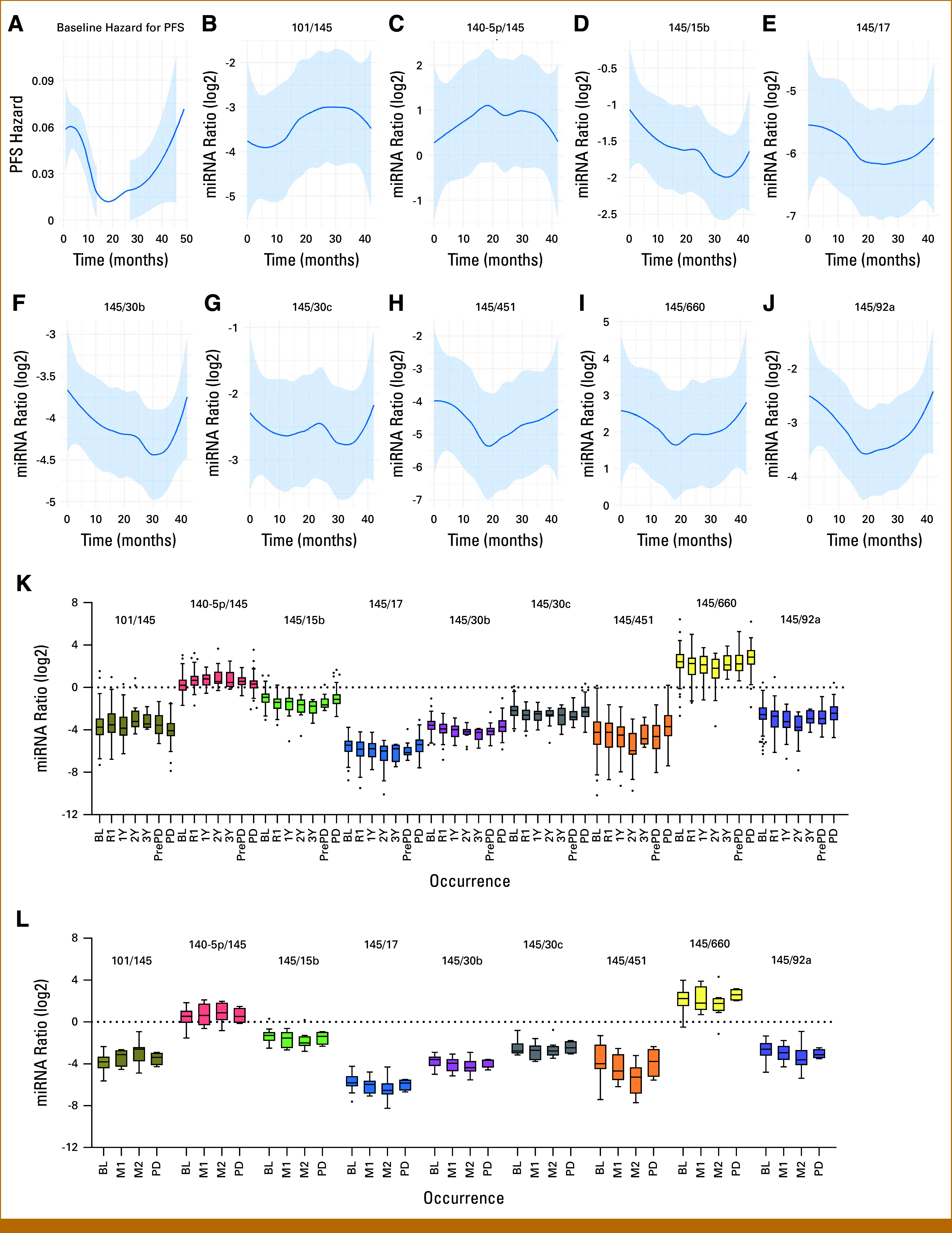
Modulation over time of (A) the PFS hazard versus (B-J) selected miRNA ratio patterns, and their distributions by longitudinal plasma sample occurrences considering (K) the 121 patients with NSCLC with longitudinal data receiving ICI in first or subsequent lines, and (L) 10 on ICI maintenance. Box plot colors represent the time of plasma sample collection: the BL; the first radiologic examination in the presence of response or stable disease (R1); annual follow-up (Y1, Y2, Y3); and the last radiologic examination before PD, PD, and on maintenance (M1, M2). BL, baseline; ICI, immune checkpoint inhibitor; miRNA, microRNA; NSCLC, non–small cell lung cancer; PD, progressive disease; PFS, progression-free survival.

Figure 3A illustrates the PFS hazard curve over time, showing a trend comparable with the longitudinal patterns of the nine selected miRNA ratios (Figs 3B-3J). Figure 3K displays the distribution of the identified miRNA ratios over time at different time points using a grouped box plot. The longitudinal modulation of miRNA ratios follows a pattern consistent with PD. Specifically, when miR-145-5p is in the numerator, the longitudinal trend follows a U-shape, with ratio values decreasing at the time of response and increasing at progression. When miR-145-5p is in the denominator, the pattern follows a reverse U-shape, reinforcing the predominant role of miR-145-5p compared with the other miRNAs in the ratio.

In an additional cohort of 10 patients with NSCLC receiving ICI as maintenance therapy after chemotherapy-induced disease stabilization, a similar trend was observed in 32 longitudinally collected plasma samples (Appendix Table A4). As shown in Figure 3L, these nine ratios exhibited the same U-shaped modulation, and the β1 values were consistent with those observed in the primary cohort (Appendix Table A5).

### The Modulation Over Time Was Consistent With the Radiologic Response

**FIG 4.**
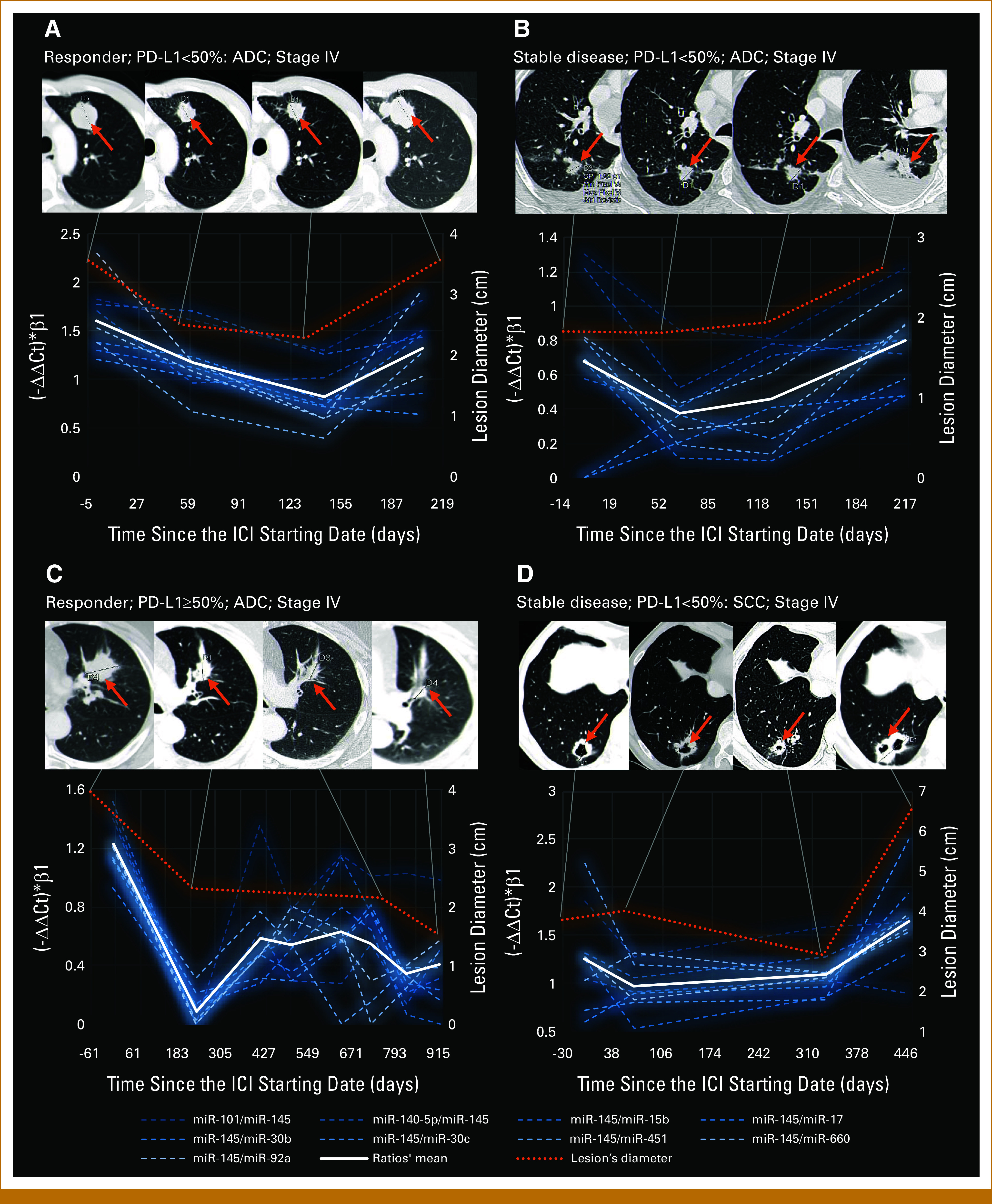
Representative images describing the longitudinal modulation of nine selected microRNA ratios, as well as their average value (ratio's mean), in four patients with lung ADC or SCC who received a benefit from treatment with immune checkpoint inhibitors. (A and B) Two patients with low (<50%) and (C and D) two patients with high (≥50%) PD-L1 expression were selected. The diameter of the main lung lesion (orange arrow) was considered as the parameter of the radiologic response. For each ratio, the –ΔΔCt values were calculated using the lowest expressor as a calibrator and multiplied by the corresponding β1 coefficient identified by the generalized estimating equation model. ADC, adenocarcinoma; SCC, squamous cell carcinoma.

Figure 4 provides representative images showing the modulation of the nine selected miRNA ratios, compared with radiologic images. To better visualize this comparison in the plots, –ΔΔCt values were calculated using the lowest expressor as a calibrator. Each –ΔΔCt value was then multiplied by the corresponding β1 coefficient identified by the GEE model, ensuring a U-shape representation even for ratios where miR-145-5p was in the denominator. Finally, the average of the nine selected miRNA ratios was calculated for each time point in each individual patient. Indeed, in both responders and patients with stable disease, miRNA ratios demonstrated a modulation pattern consistent with the radiologic response, evaluated as the largest diameter of the main lung lesion.

### MicroRNAs as Independent Prognostic Variables at Baseline

To assess whether identified miRNA ratios might have an independent prognostic value, we fitted univariate and multivariate Cox regression models to estimate the association with OS and PFS at BL. Considering the whole cohort of patients with 211 NSCLC, BL values of miR-140-5p/miR-145, miR-145/miR-15b, miR-145/miR-30b, miR-145/miR-30c, miR-145/miR-451, and miR-145/miR-92a were found to be associated with OS in univariate analysis (Appendix Table A6), but not with PFS. Moreover, except for the first two ratios, they were also found to be associated with OS in the multivariate model including clinical and biochemical characteristics (Appendix Table A7).

## DISCUSSION

In the present study, we describe the modulation of plasma miRNA profiling in patients with advanced NSCLC according to the response to immunotherapy. Thanks to the Apollo observational study, we were able to analyze a consecutive series of 211 patients with NSCLC receiving ICI therapy to assess the expression of 24 lung cancer–related circulating miRNAs in a prospective manner. Over 450 plasma samples were prospectively profiled at regular time points. For all patients, annotated clinical, molecular, and radiologic data were also available at BL. Our comprehensive data indicate that it was possible to define a mathematical model for the identification of features, which can be used to monitor response to ICI treatment.

To estimate the association between miRNA ratio modulation over time and the probability of PD, we used a two-stage statistical approach using GEE and joint models. We began by applying GEE as an initial screening tool because of its robustness in providing population-averaged estimates, even when the correlation structure is mis-specified. This characteristic ensures unbiased results in complex longitudinal data settings.^36^ Moreover, to further refine our selection, we used Joint models to explicitly account for the dependency between longitudinal changes in miRNA ratios and PFS, in cases of nonrandom dropout.^38^ Acknowledging the exploratory nature of our study, we chose to maintain the FDR threshold at 10% to allow the discovery of a broader set of miRNA ratios that may warrant further investigation.

The analysis identified nine miRNA ratios significantly associated with PD, with miR-145-5p emerging as a common feature across all of them. We have previously reported that miR-145-5p is expressed by stromal cells, including fibroblasts, macrophages, and neutrophils.^24^ Compelling evidence indicates that miR-145-5p plays a crucial role in fibroblast activation, differentiation, and extracellular matrix deposition via the KLF4 axis, linking it to processes of tissue remodeling and fibrosis.^39^ In addition to its role in fibroblast biology, miR-145-5p is actively involved in immunosuppressive signaling pathways, including transforming growth factor-β modulation, suggesting its function as a key orchestrator of immune-stromal interactions.^27,40^ More recently, miR-145-5p has been identified as a central component of the lncRNA-miRNA-CHAF1B regulatory axis, where it influences immune checkpoint regulation and tumor progression.^41^ Given these interactions, it is plausible that immunotherapy-induced changes in the TME could influence circulating miR-145-5p levels, potentially explaining, at least in part, the associations observed in our study.

When considering the BL data of all 211 patients with NSCLC, clinical variables, such as the presence of liver metastases or the blood cell count, only marginally explained the variation in miRNA ratios featuring miR-145-5p in plasma. On the other hand, six of the nine ratios associated with treatment response were also significantly linked to OS in multivariate analysis.

To more accurately assess whether the longitudinal modulation of these miRNA ratios was influenced by treatment history, we incorporated the line of therapy as an adjustment variable in our joint model. This analysis confirmed that the temporal trends of the selected miRNA ratios remained consistent across different treatment lines, indicating that their modulation was not merely a reflection of first-line versus subsequent-line therapy effects. Furthermore, these trends were similarly observed in patients receiving ICI maintenance therapy after chemotherapy-induced disease stabilization, reinforcing the hypothesis that these miRNA dynamics reflect intrinsic biological mechanisms rather than being driven by previous treatment response. Taken together, these findings support the potential of a focused panel of miRNAs, particularly miR-145-5p, as independent biomarkers for tracking response to ICI across different clinical settings.

Other circulating biomarkers have shown a similar trend over treatment. The dynamics of the peripheral expansion of specific T-cell clones, defined by their TCR repertoire, was shown to be useful for determining which patients would benefit from ICI.^13,16^ Indeed, the productive frequency of clones recognizing tumor neoantigens increased in the response-to-treatment phase compared with BL and then decreased at the time of PD. This was also reflected in the ctDNA analyses, which showed a dynamic trend inversely correlated with that of the TCR repertoire.^16^ These findings were corroborated by further evidence of a rapid decrease in ctDNA levels in patients with lung and bladder cancers who respond to immunotherapy.^42,43^ Other studies, characterizing peripheral immune cells by flow cytometry, reported an increase in PD-1+ CD8 and central memory CD4 T cells and a decrease in naïve CD4^+^ T cells and PD-L1+ natural killer cells after treatment initiation in patients with clinical benefit.^44-46^ Even soluble markers of systemic inflammation such as the C-reactive protein and interleukin-8 and circulating extracellular vesicles recently emerged as promising biomarkers to monitor the response to ICIs.^46-48^ All these studies analyzed a limited number of patients and time points, and data need to be validated in large clinical studies.

The main limitation of miRNA-based tests in liquid biopsies is their sensitivity to the nonspecific release of these small molecules because of the lysis of hematopoietic cells. Unlike the issue of clonal hematopoiesis in ctDNA analysis, which has a physiological basis, elevated levels of hemolysis are primarily caused by mechanical factors.^33^ Therefore, particular attention to collection procedures, from the time of blood withdrawal to plasma separation, is always necessary. Although this study is exploratory, given its clinical implications and since many miRNAs are actually unaffected by hemolysis (including miR-145-5p),^33^ we opted not to exclude any samples from the analysis because of hemolysis to avoid reducing the sample size and limiting the findings to a subgroup of samples.

Overall, our results contribute to the growing body of evidence supporting the use of minimally invasive biomarkers for personalized cancer treatment strategies. We posit that the complex dynamic evolution of the tumor and its microenvironment under the pressure of immune treatment can be better described by the development of comprehensive and adaptable biomarkers able to capture evolving features. Our efforts will go in this direction, where the incorporation of machine learning and artificial intelligence tools for predictive modeling of data collected from different sources holds significant promise.^30,49^ Further validation and integration of miRNA-based markers into clinical practice could enhance patient selection and improve treatment outcomes in patients with NSCLC receiving ICIs.

## Data Availability

The data will be accessible to researchers for research purposes upon signing a Data Transfer Agreement. Interested parties can request the data by e-mailing the corresponding author with a brief explanation. Requests for commercial purposes may be denied.

## APPENDIX 1. PLASMA SAMPLE COLLECTION AND MICRORNA PROFILING

At each time point, 10 mL of whole blood was collected in Vacutainer tubes with spray-coated K2EDTA and stored at room temperature. To minimize the hemolysis, leading to an unspecific microRNA (miRNA) release, plasma was separated within 2 hours by two centrifugation steps at 1,258 × *g*, 10 min, and 4°C and stored at –80°C. The level of hemolysis was measured using a NanoDrop 2000c (Thermo Fisher Scientific) spectrophotometer in the UV-Vis configuration with the baseline correction settled at 750 nm and a path length of 10 mm as by the standard protocol. High levels of hemolysis were considered for samples with an absorbance ratio 414 nm/375 nm of >2.

Within 1 week of sample collection, total RNA was extracted from 200 μL of plasma using a Maxwell RSC miRNA Tissue kit (Promega, Madison, WI) for automated nucleic acid purification and eluted in 50 μL of buffer. The plasma miRNA profile was evaluated by reverse transcription-quantitative polymerase chain reaction (RT-qPCR) using 384-well custom TaqMan Array MicroRNA Cards (Thermo Fisher Scientific) or miRCURY LNA plates (Qiagen) with endogenous controls (eg, hsa-miR-16-5p) and spike-ins to control plates, RNA extraction, and RT as per the manufacturer’s instruction. The workflow of the analysis followed our standard operating procedures. Each card or plate contained probes for the miRNAs of interest, spotted in duplicates to allow for the simultaneous analysis of eight samples: hsa-miR-101-3p, hsa-miR-106a-5p, hsa-miR-126-3p, hsa-miR-133a-3p, hsa-miR-140-3p, hsa-miR-140-5p, hsa-miR-142-3p, hsa-miR-145-5p, hsa-miR-148a-3p, hsa-miR-16-5p, hsa-miR-15b-5p, hsa-miR-17-5p, hsa-miR-197-3p, hsa-miR-19b-3p, hsa-miR-21-5p, hsa-miR-221-3p, hsa-miR-28-3p, hsa-miR-30b-5p, hsa-miR-30c-5p, hsa-miR-320a-3p, hsa-miR-451a, hsa-miR-486-5p, hsa-miR-660-5p, and hsa-miR-92a-3p. The quantitative real-time PCR reaction was performed using the ViiA7 Real-Time PCR System (Thermo Fisher Scientific). Cards or plates where the endogenous control failed or spike-in values deviated from the expected range were repeated.

**TABLE A1.** MicroRNA Ratios Associated With Liver Metastases (met) at Baseline According to the *t* Test, Using a 10% False Discovery Rate Threshold

MicroRNA Ratio	Sample Size	q Value	No Liver met	Liver met	Fold Change
miR-21/miR-30b	211	0.032	0.21	0.32	0.67
miR-21/miR-30c	211	0.032	0.55	0.78	0.71
miR-30b/miR-320	211	0.032	0.81	0.52	1.56
miR-30c/miR-320	211	0.032	0.31	0.21	1.48
miR-197/miR-21	211	0.032	0.67	0.46	1.46
miR-101/miR-30b	207	0.032	0.01	0.01	0.52
miR-101/miR-30c	207	0.032	0.02	0.03	0.54
miR-197/miR-320	211	0.032	0.12	0.08	1.52
miR-101/miR-15b	207	0.034	0.04	0.06	0.61
miR-101/miR-197	207	0.034	0.04	0.08	0.52
miR-106a/miR-30b	211	0.045	3.89	5.54	0.70
miR-140-5p/miR-30c	211	0.056	0.25	0.34	0.73
miR-106a/miR-30c	211	0.056	10.02	13.6	0.74
miR-140-5p/miR-197	211	0.056	0.67	0.94	0.71
miR-101/miR-221	207	0.057	0.02	0.04	0.60
miR-140-5p/miR-30b	211	0.057	0.10	0.14	0.70
miR-148a/miR-197	208	0.064	0.12	0.18	0.69
miR-148a/miR-30c	208	0.064	0.05	0.06	0.72
miR-101/miR-17	207	0.064	0.001	0.002	0.68
miR-30b/miR-92a	211	0.064	2.04	1.37	1.49
miR-148a/miR-30b	208	0.064	0.02	0.03	0.69
miR-101/miR-126	207	0.064	0.003	0.005	0.63
miR-19b/miR-30b	211	0.065	2.75	4.13	0.67
miR-19b/miR-30c	211	0.065	7.10	10.12	0.70
miR-17/miR-30b	211	0.065	3.96	5.38	0.73
miR-106a/miR-197	211	0.065	27.19	37.93	0.72
miR-142-3p/miR-197	210	0.065	8.22	10.57	0.78
miR-15b/miR-21	210	0.065	0.78	0.62	1.26
miR-17/miR-30c	211	0.066	10.2	13.21	0.77
miR-101/miR-140-5p	207	0.066	0.06	0.09	0.70
miR-30c/miR-92a	211	0.071	0.79	0.56	1.42
miR-21/miR-221	210	0.076	0.79	1.03	0.77
miR-101/miR-28-3p	207	0.076	0.09	0.14	0.60
miR-101/miR-140-3p	207	0.076	1.25	1.73	0.72
miR-142-3p/miR-30c	210	0.076	3.03	3.75	0.81
miR-30b/miR-660	210	0.076	67.93	43.97	1.54
miR-197/miR-19b	211	0.079	0.05	0.04	1.47
miR-30c/miR-660	210	0.079	26.34	17.69	1.49
miR-142-3p/miR-30b	210	0.079	1.18	1.51	0.78
miR-101/miR-142-3p	207	0.080	0.01	0.01	0.66
miR-101/miR-92a	207	0.082	0.01	0.02	0.71
miR-16/miR-30b	211	0.089	11.78	18.7	0.63
miR-101/miR-106a	207	0.090	0.001	0.002	0.72
miR-17/miR-197	211	0.090	27.67	36.86	0.75
miR-197/miR-92a	211	0.090	0.29	0.20	1.46
miR-126/miR-21	211	0.095	9.79	7.97	1.23
miR-145/miR-197	210	0.095	0.60	0.78	0.76
miR-145/miR-30b	210	0.098	0.09	0.11	0.76
miR-101/miR-451	207	0.098	0.004	0.006	0.66

**TABLE A2.** MicroRNA Ratios Associated With the Line of Therapy According to the *t* Test, Using a 10% False Discovery Rate Threshold

MicroRNA Ratio	Sample Size	q Value	First Line	Further Lines	Fold Change
miR-15b/miR-17	210	0.004	0.05	0.04	1.28
miR-106a/miR-15b	210	0.007	20.08	26.40	0.76
miR-486-5p/miR-92a	211	0.007	4.21	5.68	0.74
miR-16/miR-486-5p	211	0.009	5.69	4.33	1.31
miR-140-3p/miR-486-5p	211	0.013	0.002	0.001	1.39
miR-15b/miR-660	210	0.013	14.24	9.26	1.54
miR-15b/miR-486-5p	210	0.013	0.09	0.06	1.68
miR-101/miR-486-5p	207	0.013	0.003	0.002	1.56
miR-19b/miR-486-5p	211	0.013	1.37	0.98	1.41
miR-140-5p/miR-486-5p	211	0.044	0.05	0.03	1.52
miR-126/miR-15b	210	0.044	11.36	13.26	0.86
miR-142-3p/miR-660	210	0.044	97.92	67.30	1.45
miR-19b/miR-660	210	0.044	213.78	170.57	1.25
miR-106a/miR-142-3p	210	0.044	2.92	3.63	0.80
miR-142-3p/miR-486-5p	210	0.044	0.63	0.40	1.59
miR-101/miR-660	207	0.047	0.52	0.37	1.41
miR-140-5p/miR-660	210	0.048	7.78	5.68	1.37
miR-145/miR-15b	210	0.048	0.45	0.57	0.79
miR-142-3p/miR-17	210	0.049	0.33	0.28	1.21
miR-133a/miR-486-5p	206	0.049	0.004	0.002	1.79
miR-320/miR-486-5p	211	0.049	0.61	0.45	1.36
miR-21/miR-486-5p	211	0.050	0.11	0.07	1.54
miR-21/miR-660	210	0.058	17.53	12.83	1.37
miR-660/miR-92a	210	0.066	0.03	0.03	0.82
miR-142-3p/miR-145	210	0.070	15.38	12.79	1.20
miR-221/miR-486-5p	210	0.070	0.14	0.09	1.52
miR-106a/miR-140-5p	211	0.075	36.73	42.67	0.86
miR-133a/miR-660	205	0.075	0.60	0.37	1.61
miR-451/miR-486-5p	211	0.075	0.66	0.54	1.22
miR-126/miR-486-5p	211	0.075	1.04	0.70	1.49
miR-17/miR-486-5p	211	0.075	1.89	1.40	1.35
miR-15b/miR-92a	210	0.083	0.39	0.31	1.26
miR-15b/miR-19b	210	0.083	0.07	0.05	1.23
miR-15b/miR-30c	210	0.091	0.49	0.41	1.19

**TABLE A3.** The 18 Significant β1 Coefficients With SE Among 276 Generalized Estimating Equation Longitudinal Models Estimated on the Whole Cohort of 121 Patients With Lung Cancer With Longitudinal Data Treated With Immune Checkpoint Inhibitors in First and Further Lines

MicroRNA Ratio	Sample Size (events)	β1	SE	*P*	q Value
miR-101/miR-145	117 (88)	–0.3805	0.1081	<.001	0.068
miR-106a/miR-451	121 (93)	0.3520	0.1046	.001	0.076
miR-126/miR-451	121 (93)	0.2670	0.0797	.001	0.076
miR-140-3p/miR-145	121 (93)	–0.3561	0.1037	.001	0.068
miR-140-5p/miR-145	121 (93)	–0.6783	0.1943	.001	0.068
miR-145/miR-15b	121 (93)	0.8131	0.1705	<.001	<0.001
miR-145/miR-16	121 (93)	0.3960	0.1149	.001	0.068
miR-145/miR-17	121 (93)	0.5780	0.1564	<.001	0.057
miR-145/miR-30b	121 (93)	0.6698	0.1825	<.001	0.057
miR-145/miR-30c	121 (93)	0.7649	0.1734	<.001	<0.001
miR-145/miR-451	121 (93)	0.3785	0.0912	<.001	<0.001
miR-145/miR-660	120 (92)	0.4118	0.1200	.001	0.068
miR-145/miR-92a	121 (93)	0.4778	0.1399	.001	0.068
miR-148a/miR-451	121 (93)	0.3121	0.0890	.001	0.068
miR-197/miR-451	121 (93)	0.2841	0.0797	<.001	0.068
miR-19b/miR-451	121 (93)	0.4683	0.1256	<.001	0.057
miR-21/miR-451	121 (93)	0.3202	0.0896	<.001	0.068
miR-320/miR-451	121 (93)	0.3356	0.0992	.001	0.075

Abbreviation: SE, standard error.

**TABLE A4.** Clinicopathologic Characteristics of Patients With Non–Small Cell Lung Cancer Treated With Immune Checkpoint Inhibitors as Maintenance Therapy

Variable	Overall (n = 10)
Sex, No. (%)	
Male	4 (40)
Female	6 (60)
Age, years, mean (SD)	67.3 (7.5)
Smoking, No. (%)	
Never	1 (10)
Former/current	9 (90)
ECOG performance status, No. (%)	
0	5 (50)
1	5 (50)
Liver metastasis, No. (%)	
No	10 (100)
Histology, No. (%)	
Squamous	2 (20)
Nonsquamous	8 (80)
PD-L1 expression, No. (%)	
<50%	4 (40)
≥50%	6 (60)
Neutrophil-lymphocyte ratio, median (IQR)	4.6 (4-6.3)
Leukocytes, 10/µL, median (IQR)	5.9 (5.2-6.6)
Monocytes, 10/µL, median (IQR)	0.4 (0.3-0.6)
Lactate dehydrogenase, U/L, median (IQR)	358.5 (291-445)

Abbreviations: ECOG, Eastern Cooperative Oncology Group; SD, standard deviation.

**TABLE A5.** The Nine β1 Coefficients With SE of Selected microRNA Ratios Estimated by Generalized Estimating Equation Longitudinal Models on the Series of 32 Longitudinal Samples From 10 Patients With Non–Small Cell Lung Cancer Treated With Immune Checkpoint Inhibitors as Maintenance Therapy

MicroRNA Ratio	Sample Size	β1	SE	*P*
miR-101/miR-145	10	–0.8288	1.0525	.431
miR-140-5p/miR-145	10	–0.9916	0.8045	.218
miR-145/miR-15b	10	1.7516	0.7189	.015
miR-145/miR-17	10	1.4671	0.7610	.054
miR-145/miR-30b	10	4.3740	2.4789	.078
miR-145/miR-30c	10	1.6283	0.8671	.060
miR-145/miR-451	10	1.0327	0.4295	.016
miR-145/miR-660	10	0.9626	0.4289	.025
miR-145/miR-92a	10	–0.1413	0.3201	.659

Abbreviation: SE, standard error.

**TABLE A6.** Twenty-Nine of 276 microRNA Ratios Univariately Associated With Overall Survival at Baseline

MicroRNA Ratio	Sample Size	HR (95% CI)	q Value
miR-101/miR-451	207	1.18 (1.05 to 1.32)	0.029
miR-106a/miR-451	211	1.18 (1.06 to 1.32)	0.029
miR-126/miR-15b	210	1.83 (1.25 to 2.67)	0.029
miR-126/miR-451	211	1.13 (1.04 to 1.24)	0.033
miR-140-3p/miR-145	210	0.87 (0.78 to 0.97)	0.044
miR-140-5p/miR-145	210	0.79 (0.66 to 0.95)	0.044
miR-140-5p/miR-451	211	1.14 (1.04 to 1.26)	0.035
miR-142-3p/miR-145	210	0.72 (0.57 to 0.90)	0.029
miR-142-3p/miR-451	210	1.12 (1.02 to 1.22)	0.044
miR-145/miR-15b	210	1.39 (1.14 to 1.70)	0.029
miR-145/miR-16	210	1.17 (1.05 to 1.30)	0.029
miR-145/miR-21	210	1.39 (1.13 to 1.71)	0.029
miR-145/miR-221	210	1.46 (1.19 to 1.79)	0.021
miR-145/miR-30b	210	1.26 (1.07 to 1.48)	0.029
miR-145/miR-30c	210	1.30 (1.08 to 1.56)	0.029
miR-145/miR-451	210	1.15 (1.06 to 1.24)	0.029
miR-145/miR-486-5p	210	1.14 (1.03 to 1.25)	0.037
miR-145/miR-92a	210	1.24 (1.08 to 1.43)	0.029
miR-148a/miR-451	208	1.13 (1.03 to 1.24)	0.035
miR-16/miR-451	211	1.24 (1.05 to 1.45)	0.037
miR-17/miR-451	211	1.18 (1.06 to 1.32)	0.029
miR-197/miR-221	210	1.31 (1.06 to 1.63)	0.044
miR-197/miR-451	211	1.10 (1.02 to 1.19)	0.044
miR-19b/miR-451	211	1.20 (1.06 to 1.36)	0.029
miR-28-3p/miR-451	210	1.11 (1.02 to 1.21)	0.043
miR-320/miR-451	211	1.16 (1.05 to 1.29)	0.029
miR-451/miR-486-5p	211	0.73 (0.59 to 0.89)	0.029
miR-451/miR-660	210	0.80 (0.69 to 0.92)	0.029
miR-451/miR-92a	211	0.83 (0.73 to 0.96)	0.037

Abbreviation: HR, hazard ratio.

**TABLE A7.** Sixteen of 29 microRNA Ratios Showed a Multivariate Association With Overall Survival at Baseline

MicroRNA Ratio	Sample Size	HR (95% CI)^a^	q Value
miR-101/miR-451	207	1.25 (1.09 to 1.43)	0.002
miR-106a/miR-451	211	1.15 (1.01 to 1.31)	0.044
miR-126/miR-451	211	1.13 (1.01 to 1.26)	0.029
miR-140-5p/miR-451	211	1.14 (1.01 to 1.28)	0.029
miR-142-3p/miR-145	210	0.72 (0.54 to 0.97)	0.029
miR-145/miR-16	210	1.19 (1.03 to 1.37)	0.021
miR-145/miR-21	210	1.82 (1.32 to 2.44)	<0.001
miR-145/miR-221	210	1.37 (1.05 to 1.81)	0.021
miR-145/miR-30b	210	1.39 (1.09 to 1.76)	0.008
miR-145/miR-30c	210	1.40 (1.07 to 1.84)	0.011
miR-145/miR-451	210	1.14 (1.03 to 1.27)	0.011
miR-145/miR-486-5p	210	1.15 (1.02 to 1.31)	0.029
miR-145/miR-92a	210	1.31 (1.08 to 1.57)	0.005
miR-17/miR-451	211	1.51 (1.01 to 1.32)	0.044
miR-19b/miR-451	211	1.19 (1.02 to 1.39)	0.029
miR-28-3p/miR-451	210	1.11 (1.01 to 1.23)	0.044

Abbreviation: HR, hazard ratio.

^a^ Model adjusted by sex, age, smoking, line of therapy, performance status, histology, liver metastasis, PD-L1 expression, neutrophil-lymphocyte ratio, leukocytes, monocytes, and lactate dehydrogenase.
